# Iterative Regression of Corrective Baselines (IRCB): A New Model for Quantitative Spectroscopy

**DOI:** 10.1021/acs.jcim.4c00359

**Published:** 2024-06-19

**Authors:** Matthew Glace, Roudabeh S. Moazeni-Pourasil, Daniel W. Cook, Thomas D. Roper

**Affiliations:** † Department of Chemical and Life Sciences Engineering, Virginia Commonwealth University, Richmond, Virginia 23284, United States; ‡ Medicines for All Institute, Virginia Commonwealth University, Richmond, Virginia 23284, United States

## Abstract

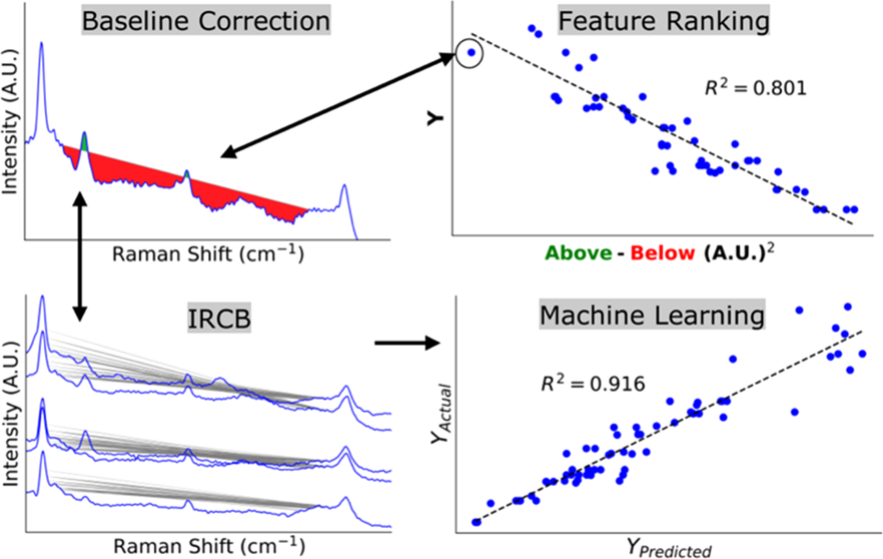

In this work, a new model with broad utility for quantitative spectroscopy development is reported. A primary objective of this work is to create a novel modeling procedure that may allow for higher automation of the model development process. The fundamental concept is simple yet powerful even for complex spectra and is employed with no additional preprocessing. This approach is applicable for several types of spectroscopic data to develop regression models that have similar or greater quality than the current methods. The key modeling steps are a matrix transformation and subsequent feature selection process that are collectively referred to as iterative regression of corrective baselines (IRCB). The transformed matrix (**X**_**transform**_) is a linearized form of the original **X** data set. Features from **X_t_**_**ransform**_ that are predictive of **Y** can be ranked and selected by ordinary least-squares regression. The best features (rows of **X_t_**_**ransform**_) are linear depictions of **Y** that can be utilized to develop regression models with several machine learning models. The IRCB workflow is first detailed by using a case study of Fourier transform infrared (FTIR) spectroscopy for prepared solutions of a three-component mixture. Next, IRCB is applied and compared to benchmark results for the 2006 “Chimiométrie” near-infrared spectroscopy (NIR) soil composition challenge and Raman measurements of a simulated nuclear waste slurry.

Spectroscopic instrumentation, when combined with chemometric or machine learning models, becomes a very effective tool for process analytical chemistry (PAC).^1,2^ These techniques are nondestructive and can be employed in-line, or online, to monitor processes in real time.^3^ Raman and infrared spectroscopies have been applied for an increasing number of use cases. Among others, these applications of spectroscopy include food,^4−6^ pharmaceuticals,^7−10^ cosmetics,^11^ tobacco,^12^ and nuclear waste.^13,14^ In 2004, the US Food and Drug Administration (FDA) and the International Council for Harmonization (ICH) established an initiative to apply process analytical technologies (PAT), including spectroscopic PAC, for manufacturing quality assurance.^15,16^ Many studies have reported on the use of spectroscopic analyzers, such as infrared (IR) and Raman, to monitor various stages of pharmaceutical manufacturing.^8,9,17−22^ Spectroscopic PAT is useful both for real-time release and model predictive control.^7,9,20,22^ The linking of PAT to continuous manufacturing for real-time optimization and control using artificial intelligence was referred to by Price et. al as the “holy grail”.^7^

Because spectrometers do not provide physical separation between the measured compounds, the resulting measurement is the combined molecular fingerprint for all of the compounds within the mixture—providing data-rich but highly complex spectra.^23,24^ Partial least-squares regression (PLS-R) and principal component analysis (PCA) have typically been utilized to deconvolute and model the resulting spectra.^25−27^ The development and implementation of quantitative models from the spectra has historically been a challenging task.^28−30^ Data treatment, known as preprocessing, is also typically required for complex mixtures to correct for nonlinearity and to focus on the model on the analyte of interest. Significant efforts have been made in the development of new preprocessing techniques to improve the capabilities of spectroscopic PAC to model more complex data, such as crude reaction mixtures. As such, new types of data processing are frequently reported, some of which rely on iterative approaches or neural networks for preprocessing optimization.^31−39^ Although artificial intelligence has previously been applied for preprocessing treatments, few examples for end-to-end automated quantitative model development have been attempted.^40^ Automated end-to-end quantitative model development may provide significant advantages for the generalizable accuracy and repeatability of chemometric models.

In this work, we introduce a new model for quantitative spectroscopy development termed iterative regression of corrective baselines (IRCB). The proposed approach is simple, intuitive, and highly automated; yet it can provide valuable spectra insights and be used to generate predictions that may outperform existing PLS-R models. The approach is based in statistics and does not rely on any spectral interpretation from molecular structure or carry forward previous knowledge. IRCB is utilized in tandem with several supervised machine learning models such as ensemble linear regression (ELR), random forest (RF) from scikit-learn,^41^ and extreme gradient boosting^42^ (XGB) to complete the model construction. While the IRCB model itself may be conceptualized as a preprocessing step for machine learning, it is used without any additional preprocessing. In summary, IRCB is an expansive matrix transformation that effectively generates many linear predictors from the original data.

We hypothesize that IRCB can improve the automatability and interpretability of regression model development for many types of spectroscopic analytical techniques. The employed computational approach can be beneficial to identify the spectral regions of high selectivity and result in more consistent results across different model developers. Here within, the effectiveness of the IRCB model is assessed with several diverse spectroscopic PAC case studies. The IRCB workflow is first detailed using a Fourier transform infrared (FTIR) spectroscopy case study for prepared solutions of propofol and two structurally related impurities.^43^ Next, IRCB is applied, and the statistical results are compared for two additional previously benchmarked case studies. Case study 2 is near-infrared (NIR) measurements of crude soil samples,^44^ and case study 3 is Raman spectroscopy quantification of solids in a slurry of simulated nuclear waste.^14^ The additional insights from the novel matrix transformation are also discussed.

## Experimental Section

### Iterative Regression of Corrective Baselines (IRCB)

**Figure 1:**
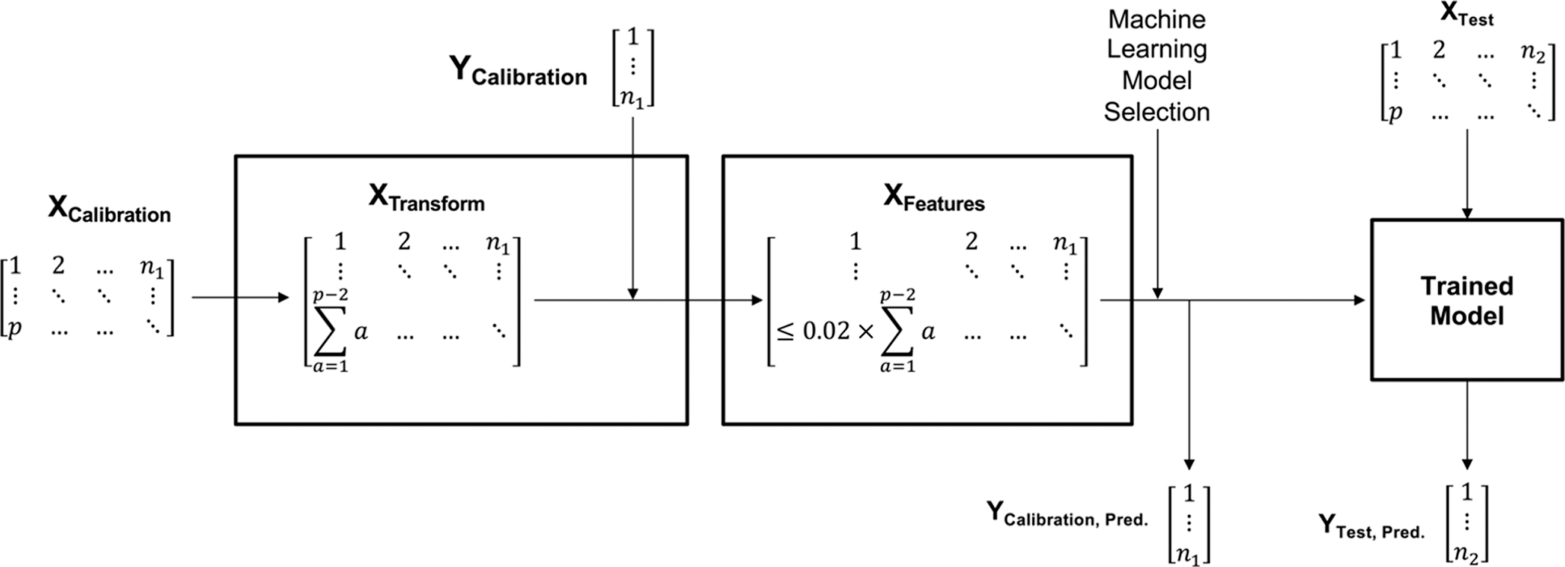
Procedure for model fitting with the IRCB.

The IRCB model is detailed in Figure 1, where “*n*” is the number of spectra and “*p*” is the number of data points in each spectrum. In addition to the calibration matrices of **X** (spectra) and **Y** (concentrations), the choice of a final machine learning predictive model is also required for regression model development. No preprocessing or structural information about the spectra is required. The necessary model parameters (in the form of baseline indices) are passed from the IRCB procedure to the trained model for application on **X**_**test**_. All test samples, even within k-folds, are excluded for the entirety of model development. The **X_t_**_**ransform**_ columns remain sample-specific, whereas the number of rows in **X_t_**_**ransform**_ along the expanded axis is a function of the original number of data points “*p*” in the spectra. The operation from **X**_**calibration**_ to **X_t_**_**ransform**_ results in a unit change of the matrix elements from arbitrary units (a.u) to (a.u.)^2^. The generation of **X_t_**_**ransform**_ is facilitated by the application of an iterative baseline correction and a subsequent area summation. Each **X_t_**_**ransform**_ entry stores the area between a spectrum as a “baseline”. Every unique baseline is a line segment with end points at two specific locations along the spectrum.^8^ The position of the line segment end points is row-specific within the **X**_**transform**_ and sufficient to describe the application of a unique baseline to all samples in **X_c_**_**alibration**_ and **X**_**test**_.

For each row of **X**_**transform**_, a unique pair of start and stop data point locations will be applied to generate a linear baseline for each spectrum of **X**_**calibration**_ individually. Because each baseline and spectra contain discrete data, a uniformly scaled area between them can be calculated using a trapezoidal summation applying a length of one between data points.^8^ For all data sets with equidistant spacing between observations, the spacing is an arbitrary scale factor, so it can be removed for computational efficiency. Therefore, the simplest area summation procedure is taking the sum of the matrix that results from subtracting the baseline from the spectral response. Accordingly, the “inverted” areas (above the spectra and beneath the corrective baseline) are considered negative during the area approximation.

If the baseline connects two adjacent data points, then the area between the baseline and the response will be zero. Therefore, only baselines spanning at least three data points result in a summed area that is nonzero and are useful for the next step of the operation. The number of potentially useful linear baselines, or the maximum number of rows contained in **X**_**transform**_, is defined as , where *p* is the number of data points in the spectra and “*a*” is an arbitrary counter variable. The baseline generation algorithm is a comprehensive approach that considers every possible linear connection of two data points that can result in a nonzero area between the linear baseline and the spectra within the range of the two data points.

Because of the expansive nature of the iterative baseline correction, **X**_**transform**_ is significantly larger than the original **X** data set. A procedure to select the most useful features (rows) of **X**_**transform**_ is next employed. Each row within **X**_**transform**_ is assigned a coefficient of determination (*R*^2^) for **Y**_**calibration**_ and sorted row-wise by the *R*^2^ value assigned. Because of this sorting, the highest rated features of **X**_**transform**_ are linear depictions of **Y**. After sorting, an arbitrary number of the top features (highest *R*^2^) within **X**_**transform**_ are carried forward into the new matrix **X**_**features**_. For the case studies described in this work, generally around 2% of the highest rated features of **X_t_**_**ransform**_ were selected for **X**_**features**_, although using less may also result in an adequate prediction, as dictated by the complexity of the system. For any model, the start and stop baseline locations from the calibration set are indexed to generate an equivalent **X**_**features**_ matrix (same number of rows) for the test set(s). The python code to produce and sort the **X**_**transform**_ matrix is from the initial spectra detailed in the Supporting Information and is available from the GitHub linked in the Data and Software Availability section. For readability, the most fundamental version of the baseline correction operation is shown in the Supporting Information, although a much faster multicore version is available from the GitHub. For the regression operation, a less resource-intensive multithreading approach was employed to significantly enhance computational feasibility, as shown in the Supporting Information.

**Figure 2:**
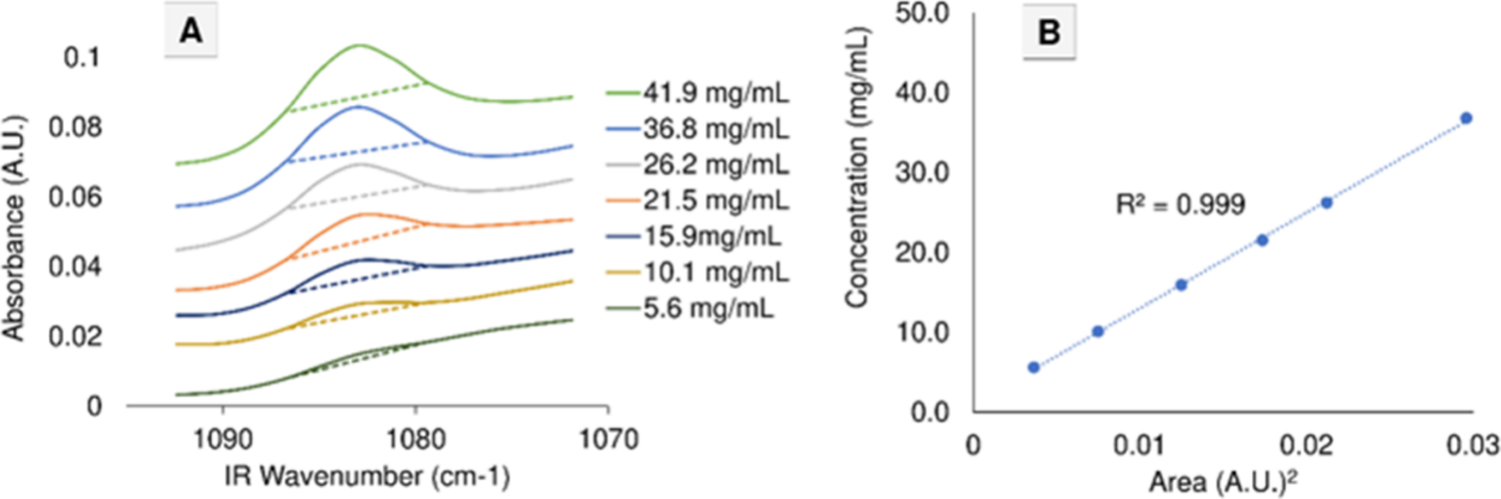
Sample baseline correction and *R*^2^ assignment for a highly effective baseline (1079–1087 cm^–1^).

In a classical spectroscopic interpretation, the simplest corrective baseline (row of **X**_**transform**_) captures a selective analyte peak which correlates to concentration as dictated by Beer–Lambert’s law. An example of a highly rated baseline is shown in Figure 2. The area between the spectral response and the baseline, which spans from 1079 to 1087 cm^–1^, is selective for the targeted component concentration (**Y**_**calibration**_) as demonstrated by a high *R*^2^ value. This indicates that this feature of **X**_**transform**_ will be useful for making predictions about **Y** and is likely to be included in **X**_**features**_. The matrix **X_f_**_**eatures**_ is useful as input for a variety of predictive machine learning models, including ensemble linear regression (ELR), random forest (RF), and extreme gradient boosting (XGB).

The predictions produced by the various machine learning models may vary in effectiveness based on the complexity of the spectra and size of the calibration data. For simple cases, such as a three-component mixture of pure compounds, ELR was the most effective and interpretable. For the ELR approach, each **X**_**features**_ element (baseline) was used as a simple linear regression model and the predictions of numerous linear regression models were averaged for the test set prediction. The exact number of ensembled regression models included was determined by minimizing the error of the cross-validation prediction of the calibration set. This ELR strategy was specifically developed for compatibility with IRCB to select the most appropriate number of baselines to include in the final predictions—up to the maximum threshold set by the user. For the more complicated examples, RF and XGB were required to produce accurate and robust test set predictions. However, for the RF and XGB models, the percentage of the sorted **X**_**features**_ that was included in **X**_**transform**_ must be manually specified by the user and is used directly without the potential reduction that is possible in the ELR case.

### Materials

Materials were used as received from vendors. 2,6-Diisopropylphenol (100%) and 2-isopropyl phenol (98%) were procured from Chem-Impex International, Inc. 4-Hydroxy-3,5-diisopropylbenzoic acid (98%) was procured from Combi-Blocks. The 4-hydroxy-3,5-diisopropylbenzoic acid used for spectroscopic measurements was synthesized in-house and purified by recrystallization from heptane. Acetonitrile (HPLC grade) was procured from Sigma-Aldrich.

### Sample Preparation—Case Study 1

Each stock solution of the analyte was generated by the dissolution of the purified compounds into acetonitrile. A total of 14 calibration and 10 test samples were prepared in the range of 5.0–45.0 mg mL^–1^ for each analyte. For reference concentrations, each sample was analyzed by HPLC in duplicate after dilution of the FTIR samples into the linear range of the HPLC calibration. The concentrations as determined by HPLC were considered the true analyte concentration (Table S1).

### High-Performance Liquid Chromatography

For each analyte (Propofol [1], 2-IP [2], HDIPBA [3]), a 6-point calibration curve, from 0.05 to 1.00 mg mL^–1^, was prepared by acetonitrile. A minimum coefficient of variation (*R*^2^ = 0.99) was enforced for all HPLC calibrations. Samples were analyzed in duplicate. Full details of the HPLC method are outlined in the Supporting Information.

### Fourier Transform Infrared Spectroscopy

A ReactIR 15 instrument was equipped with a DS Micro Flow Cell. The detector was chilled for at least 2 h with liquid N_2_ prior to analysis. FTIR samples were maintained at room temperature prior to and during analysis. Samples were analyzed by manual injection into the Micro Flow Cell DS DiComp. A 30 s scan time was selected, and three spectra were collected for all calibration and test samples.

## Results and Discussion

A primary aim of the methods employed in this study was to model the target **Y** from the multicomponent calibration set **X** without reference spectra, manual peak identification, or inferred structural knowledge. To demonstrate the wide versatility and usefulness of IRCB, three case studies were investigated and reported. An outline of all three case studies is given in Table 1. The three case studies utilized different instruments (FTIR, NIR, Raman) and were applied to physically different sample types (Solution, Solids, Slurry). Similarly, **X_f_**_**eatures**_ was used as an input for three different machine learning models to generate the final **Y** prediction. In case study 1, the solution concentration of three different pharmaceutically relevant analytes was predicted using FTIR. In case study 2, IRCB was applied to a previously reported data set that utilized NIR to measure solid soil samples. Lastly, in case study 3, IRCB was tested to predict the concentrations of five solids within a complex slurry using Raman spectroscopy. For case studies 2 and 3, the modeling results may be compared to the previously published benchmarks.^14,44^

**Table 1:** Outline of the Case Studies

case study	description	num. models	instrument	type	model
1	propofol	3	FTIR	solution	ELR
2	soil	3	NIR	solids	RF
3	nuclear waste	5	Raman	slurry	XGB

### Case Study 1: FTIR for Three-Component Mixture

**Figure 3:**
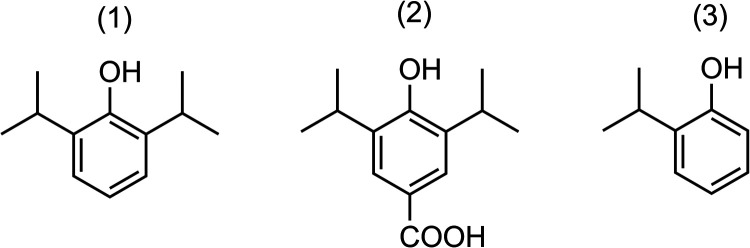
Chemical structures for (1) 2,6-diisopropylphenol (Propofol), (2) 4-hydroxy-3,5-diisopropylbenzoic acid (HDIPBA), and (3) 2-isopropyl phenol (2-IP).

In the first case study, the model development procedure for a three-compound mixture of propofol and two structurally similar impurities is detailed. The three chemical structures from the solution are shown in Figure 3. These three compounds were chosen based on their structural similarity. Propofol has no unique functional selectivity when compared to those of 4-hydroxy-3,5-diisopropylbenzoic acid (HDIPBA) and 2-isopropyl phenol (2-IP) collectively.

**X**_**calibration**_ contained 42 calibration spectra (14 independent samples × 3 replicates) and 1798 data points for each spectrum. The samples all contained each of the three analytes at concentrations between 5.0 and 45.0 mg mL^–1^. The matrix transformation was applied to create **X**_**transform**_ of size 1,613,706 × 42. Although three different analyte models were developed, **X**_**transform**_ is a comprehensive matrix of **X_c_**_**alibration**_ that is generic to all three substrates. An **X_f_**_**eatures**_ matrix must be generated for each substrate independently using the generic **X**_**transform**_ and a substrate-specific **Y_c_**_**alibration**_. The top four baselines of **X**_**features**_ are described for each of the three substrates in Table 2. Highly selective baselines were discovered for each analyte as demonstrated by several *R*^2^ values >0.99. The upper and lower limits describe the end point locations of the baselines contained within **X_f_**_**eatures**_. Notably, Table 2 describes only the four most selective baselines for each of the three substrates, but **X**_**features**_ contains many additional baselines for each analyte with a gradually decreasing *R*^2^ value for each entry. A full description of each baseline in the **X_f_**_**eatures**_ matrices is available for this case study and the others in the Supporting Information.

**Table 2:** Best Baselines for Case Study 1

start (cm^–1^)	stop (cm^–1^)	*R*^2^	slope (A.U.^2^ mg^–1^)	intercept (A.U.^2^)
a. Propofol				
1443	1650	0.998	–3.50 × 10^01^	–2.95 × 10^02^
1201	1214	0.998	8.03 × 10^02^	–4.10 × 10^00^
930	975	0.998	–4.07 × 10^02^	–1.03 × 10^02^
1443	1648	0.998	–3.57 × 10^01^	–2.96 × 10^02^
b. HDIPBA				
1655	1778	0.999	2.73 × 10^01^	2.04 × 10^00^
1655	1782	0.999	2.73 × 10^01^	2.22 × 10^00^
1655	1780	0.999	2.73 × 10^01^	1.87 × 10^00^
1648	1788	0.999	2.65 × 10^01^	4.46 × 10^00^
c. 2-IP				
1079	1087	0.999	1.19 × 10^03^	1.05 × 10^00^
831	1225	0.999	–6.16 × 10^00^	4.40 × 10^01^
1081	1085	0.999	7.96 × 10^03^	4.85 × 10^–01^
1497	1517	0.999	4.24 × 10^02^	5.12 × 10^00^

**Figure 4:**
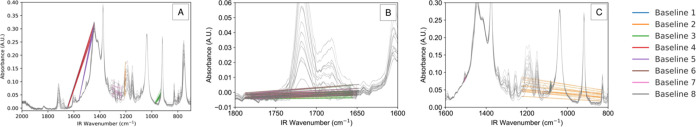
Plot of top baselines in case study 1 for (A) propofol, (B) HDIPBA, and (C) 2-IP.

In Figure 4A–C, the best eight baselines were plotted onto the calibration sets for propofol, HDIPBA, and 2-IP, respectively. Each of the baselines in Table 2 (and four more for each analyte) is overlaid to the spectra of Figure 4A–C in a unique color. Several of the baselines selected by the IRCB are directly indicative of functional selectivity. In 2-IP, aromatic C–H bending near 1500 and 1080 cm^–1^ was captured. The carboxylic acid functional group near 1725 cm^–1^ for HDIPBA was similarly selected. Additionally, several nonintuitive baselines are shown to be quantitative concentration indicators.

Although propofol lacks functional group selectivity, the resulting **X**_**features**_ elements from several baselines were still highly correlated with **Y**. The selection of the best baselines by the IRCB model is a comprehensive approach that does not require any structural knowledge or manual interpretation because every possible linear baseline is tested. Those selected by the protocol are unbiased by a portion of the spectra that the operator may be predisposed to believe is the most selective. The selected baselines are considered the best only because when applied to the calibration set, they result in areas that have the highest correlation to the targeted **Y**.

In some cases, the best baseline regions for different analytes may cross or entirely overlap. For example, the selective 2-IP baseline from 1499 to 1517 cm^–1^ is entirely contained within the selective propofol baseline from 1445 to 1562 cm^–1^. The baseline discovery process may be enhanced by the area summation feature that allows “inverted” portions to be considered negative. For example, the second highest rated 2-IP baseline from 831 to 1225 cm^–1^ has significant spectral responses both above and below the corrective baseline. However, the sum of positive and negative areas balances to give a linear response in the overall area of the baseline to the 2-IP concentration. In some instances, the applied baseline resulted in a net negative area of the spectrum being captured. It can be seen in the best propofol baselines that some selective regions (ex. 1443–1650 cm^–1^) are entirely “inverted”. In terms of classical spectroscopic interpretation, this result was initially perplexing. However, every typically “clean” baseline for a unique selective peak is contained within **X**_**transform**_ and the complex solutions appearing within **X**_**features**_ demonstrated higher correlation with **Y** than the simpler ones that may be easier to select manually.

The selection and application of a machine learning predictive model are required for converting the **X**_**features**_ in **Y** prediction for the calibration and test sets. For case study 1, ensemble linear regression (ELR) was applied to create the regression model. Using ELR, each row in **X**_**features**_ served as a linear regression model that was averaged into the final prediction for **Y**. The number of linear regression models to average for the final ELR prediction was determined by minimizing the 5-fold cross-validation error of the calibration set. The number of ensemble regressions included was 98, 29, and 8 for propofol, HDIPBA, and 2-IP, respectively. For computational efficiency, the maximum percentage of **X**_**transform**_ that may be included in **X**_**features**_ must be manually specified. However, with the ELR approach, the number of features selected can be automatically reduced below the user-specified maximum threshold. For the later RF and XGB models, the user-specified threshold percentage was used directly without the potential reduction.

**Figure 5:**
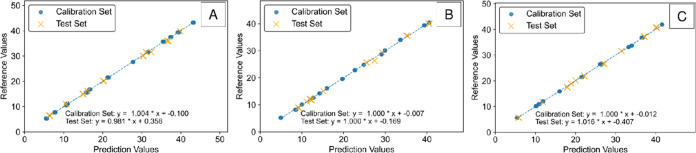
Case study 1 regression models plotted for (A) propofol, (B) HDIPBA, and (C) 2-IP.

For this case study, the IRCB-ELR model was applied to a selection of 30 spectra (10 samples x 3 scans) for an external test set evaluation. For the test set, **X_f_**_**eatures**_ for each analyte was generated by using the corresponding baseline indices from the calibration set. The statistical results for each of the three analytes are outlined in Table 3a. The model provided an excellent fit for each of the three target analytes as indicated by a test set *R*^2^ of >0.99 and a test RMSE of <0.50 mg mL^–1^. The results indicate that the baselines selected by assigning an *R*^2^ value to the calibration set samples were effective for predicting the concentration of the test set. The results show that the IRCB model is effective because the baselines selected by the model are directly useful to generate a quantitative test set prediction. The modeling results are plotted in Figure 5A–C for propofol, HDIPBA, and 2-IP respectively.

**Table 3:** Statistical Results for (a) Case Study 1 and (b) Case Study 2^a^

a. Statistical results for case study 1. Propofol system using ensemble linear regression (ELR) for FTIR							
	RMSE				*R*^2^		
componet	units	calibration	C.V	test	calibration	C.V.	test
propofol	mg mL^–1^	0.189	0.199	0.452	1.000	1.000	0.999
HDIPBA	mg mL^–1^	0.210	0.248	0.457	1.000	0.999	0.998
2-IP	mg mL^–1^	0.207	0.238	0.323	1.000	1.000	0.999

b. Statistical results for case study 2. Crude soil samples using random forest (RF) with NIR							
	RMSE				*R*^2^		
component	units	calibration	C.V	test	calibration	C.V.	test
nitrogen	g kg^–1^	0.185	0.582	0.657	0.977	0.756	0.766
carbon	percent	0.324	0.903	0.527	0.970	0.762	0.880
CEC	meq 100 g^–1^	1.597	6.643	3.739	0.944	0.000	0.715

a CEC = Cation exchange capacity, HDIPBA = 4-hydroxy-3,5-diisopropylbenzoic acid, 2-IP = 2-isopropyl phenol, C.V. = Cross-Validation.

### Case Study 2: “Chimiométrie 2006” Soil Quantification with NIR Spectroscopy

Crude samples can introduce significant spectral complexity as compared to prepared solutions with limited interfering analytes. As such, for case study 2, IRCB was next tested using the “Chimiométrie 2006 Conference” soil quantification challenge.^44^ This data set contains NIR measurement of 618 soil samples and offline measurements for total nitrogen (g kg^–1^ dry soil), carbon percentage in dry soil (carbon, %), and cation exchange capacity (CEC, meq 100 g^–1^ of dry soil).^44^ The external test set was utilized as outlined by the conference guidelines.^44^

Although this data set was collected over a multiday period with likely instrument and environment variation, no preprocessing was performed prior to the IRCB deployment. The baseline correction was applied to generate **X**_**transform**_ from the raw data. Next, iterative regression was performed on **X**_**transform**_ to generate the three **X**_**features**_ matrices for the three **Y**_**calibration**_ matrices (nitrogen, carbon, and CEC). For this case study, each **X**_**features**_ matrix retained the top 2% of **X**_**transform**_ that was most selective for the respective **Y**_**calibration**_. As previously mentioned, the percentage of **X**_**transform**_ that is included in **X**_**features**_ must be manually specified for the ICRB-RF model. The effect of this percentage for case study 2 is shown in Figure S2. Generally, the performance began to plateau at around 2% inclusion.

**Figure 6:**
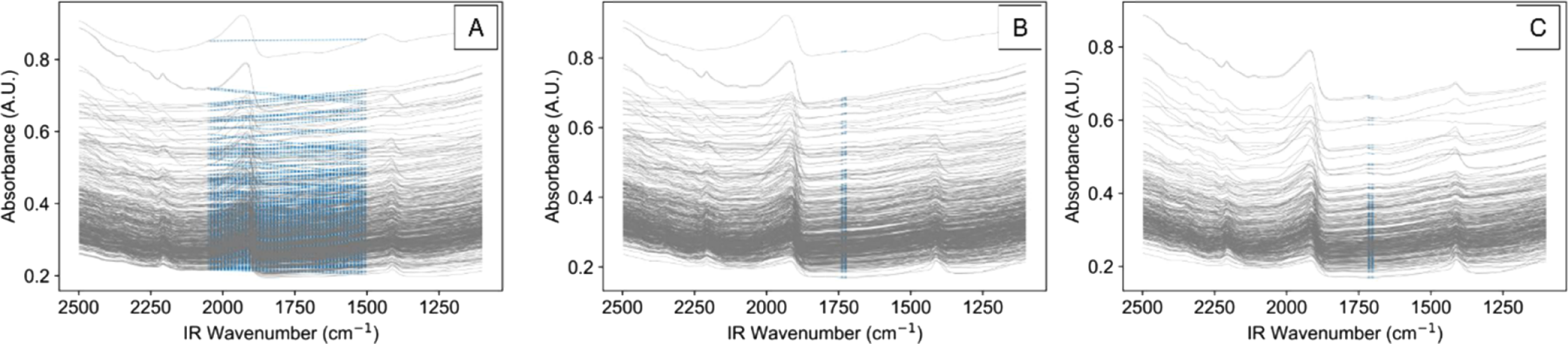
Plot of top baselines in case study 2 for (A) nitrogen, (B) carbon, and (C) CEC.

The best baseline for each component is plotted in Figure 6A–C for nitrogen, carbon, and CEC, respectively. The full **X**_**features**_ for each component is available in the Supporting Information. The top baselines for the three targeted **Y**_**calibration**_ matrices in case study 2 were significantly less predictive than those reported for the simpler system in case study 1. The top *R*^2^ values for the best individual baselines to **Y**_**calibration**_ were 0.701, 0.667, and 0.509 for nitrogen, carbon, and CEC, respectively. These *R*^2^ values represent the coefficient of determination for the top row of each **X**_**features**_ matrix with respect to its respective **Y**_**calibration**_. One clear advantage of the outlined approach is the ability to determine which portion of the spectra is most correlated to the **Y** of interest. For example, cation exchange capacity (CEC) does not directly correspond to a known functional group, but using the IRCB model, it can be observed that 1698–1720 cm^–1^ is a region of key interest (Figure 6C). The best nitrogen baseline (Figure 6A) was complex, as it contained both positive and negative regions that summed to give a linear (*R*^2^ = 0.701) response.

**Figure 7:**
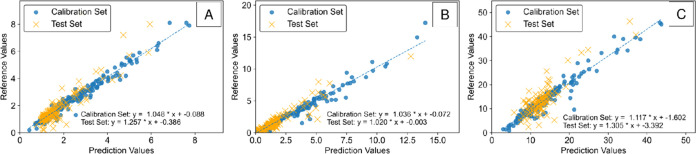
Case study 2 regression models plotted for (A) nitrogen, (B) carbon, and (C) CEC. Random forest (RF) model with default hyperparameters as described in Table S2.

Given that best baselines were not immediately highly effective regression models for the target analytes, it was hypothesized that a nonlinear machine learning model would be useful to generate more robust predictions. As such, random forest (RF) machine learning was selected to generate regression predictions from the **X_f_**_**eatures**_ matrices. The **X**_**features**_ matrices for the calibration set were used to train RF models from scikit-learn.^41^ The statistical results for the case study 2 IRCB-RF regression models are outlined in Table 3b. The model predictions and true values are plotted in Figure 7 for both the calibration and test sets with the default RF hyperparameters, as shown in Table S2. The IRCB-RF NIR soil composition test set prediction statistical parameters ranked highly among the six previously reported models and statistical comparison of the benchmarked test set is shown in Table S3.^44^ Although the test set fitting for the “CEC” model was considered satisfactory compared to other analyses of this data set, the cross-validation error was quite high. It is hypothesized that this is due to outliers within **Y**_**calibration**_ for this data set. The cross-validation statistics for other models were not previously reported.^44^

Despite test set predictions, they were extremely competitive with other approaches (Table S3), overfitting of the RF model remained problematic as indicated by the difference in RMSE values between the calibration and test sets (Table 3b). In the next model development iteration, the RF hyperparameters were tuned to address overfitting by minimizing the RMSE of 5-fold cross-validation of the calibration set. An exhaustive grid search approach was utilized for the RF hyperparameter design space shown in Table S2. The design space of the grid search was made to be more conservative than the default hyperparameters by implementing limitations, such as increasing the minimum samples per leaf and per split. The resulting models and the selected best hyperparameters are shown in Table S4. This approach did reduce the absolute difference in the RMSE between the test and calibration sets as compared with default RF hyperparameter values but typically did not benefit the statistical metrics of the test set. As an exception, the CEC test set *R*^2^ was improved from 0.715 (Table 3) to 0.746 (Table S4).

In summary, the original **X** is transformed into a novel matrix form using IRCB—that is a more linearized depiction of the original data. After the best **X**_**features**_ values are identified using IRCB, the RF model is then able to weight linear predictors and secondary interactions within **X**_**features**_. The overall regression prediction of the RF model surpasses the predictive capacity of any one baseline region. For example, in the carbon model, the best-fitted baseline to **Y**_**calibration**_ was *R*^2^ = 0.667 but by using the best 2% of baselines (**X**_**features**_) with RF, the overall prediction for an unknown test set was *R*^2^ = 0.880 (Table 3b). This indicates that significant predictive power is being generated within **X**_**transform**_ and effectively extracted into **X**_**features**_ using the outlined iterative regression sorting procedure. In summary, the baselines that are highly correlated to the calibration set are predictive of test set concentrations. It is shown within the results of case study 2 that IRCB can find selective responses even for complex mixtures with overlapping signals. This is further demonstrated by the containment of the best carbon and CEC baselines within the best nitrogen baseline (Figure 6).

### Case Study 3: Raman Spectroscopy for Dense Slurry Solid Quantification

In case study 3, the IRCB approach was applied to another previously published data set, which utilized Raman spectroscopy to measure a dense multicomponent slurry designed to simulate nuclear waste.^14^ The objective of the model reported is to predict the solid concentration for each of the five analytes (kyanite, wollastonite, olivine, silica, and zircon) using 66 spectra of the slurry. This data set provides additional complications compared to the previous two as the Raman probe is exposed to both solids and liquids. Moreover, the composition of the solids exposed to the probe may fluctuate as the slurry is stirred. In the original work,^14^ five solid analytes were modeled using partial least-squares regression (PLS-R) and assessed using leave-one-out cross-validation (LOOCV). For our approach, a 10-fold cross-validation was applied to develop and assess the model of each analyte.

**Figure 8:**
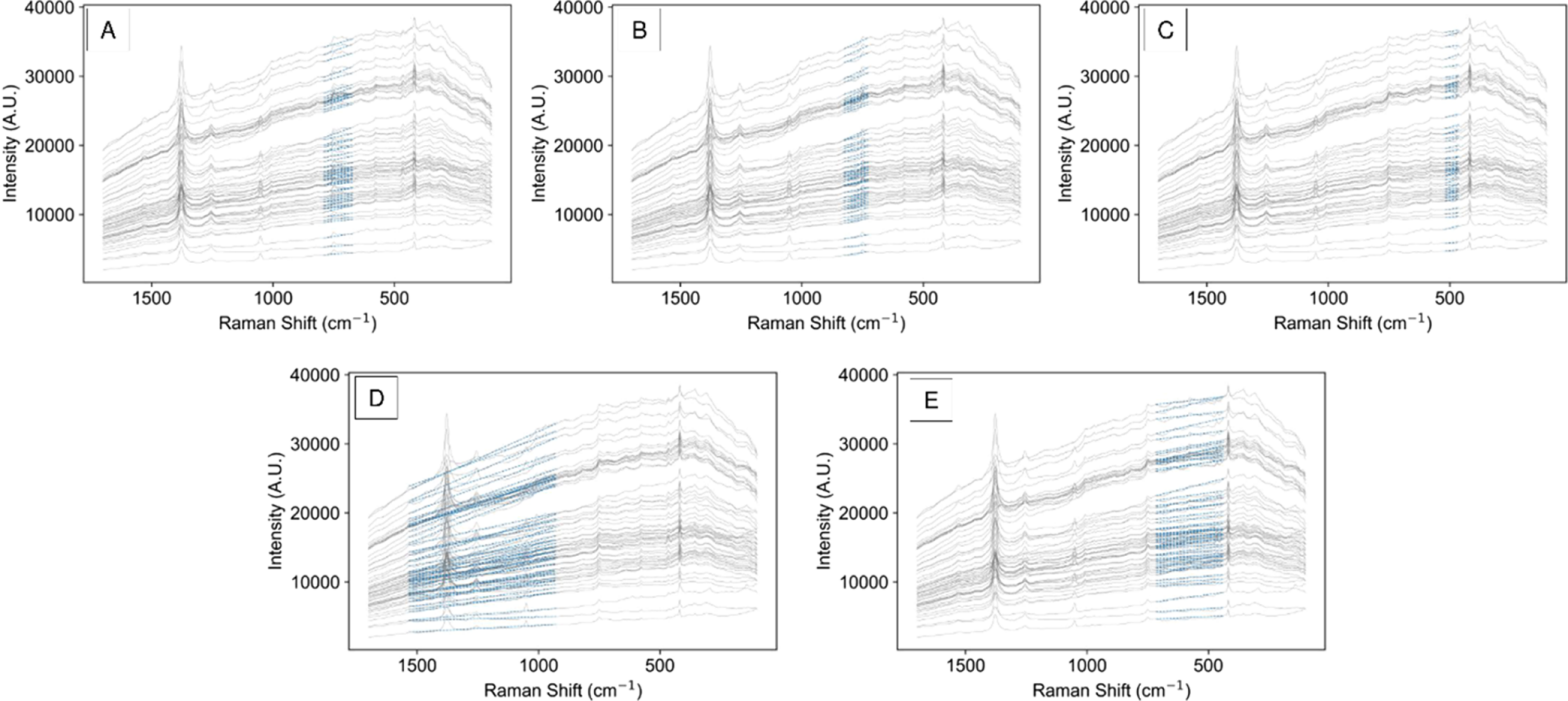
Plot of top baselines in case study 3 for (A) kyanite, (B) wollastonite, (C) olivine, (D) silica, and (E) zircon.

First, the generic **X**_**transform**_ was generated and then **X**_**features**_ were generated for each analyte within each cross-validation fold. Once again, **X**_**features**_ consisted of the top 2% of **X**_**transform**_ for each analyte within each fold. For fold-1, the top baseline for each component is plotted in Figure 8. Although the results are shown from the first fold, the order and statistics of the top baselines varied only slightly across the k-folds. It can be observed in Figure 8 that the most selective baseline for each analyte is far more complex than a single analyte peak. For almost all of the samples, there are portions of the spectral response both above and below the baselines that sum to increase the linearity of the response. Given this complexity, the manual identification of selective regions is extremely challenging or entirely impossible. This evidence helps to support our hypothesis that IRCB is beneficial for creating a standard automatable approach.

A machine learning package, XGBoost (XGB), which stands for extreme gradient boosting, was used to develop the final regression model for each fold separately using the **X_f_**_**eatures**_ matrices. XGB is a powerful machine learning model that uses gradient boosted decision trees to solve many types of supervised regression and classification problems.^42^ While RF may also be suitable for this case study, XGB is also an acceptable choice and further demonstrates the versatility of IRCB with several machine learning packages. For each IRCB-XGB model (specific to the analyte and fold), XGB hyperparameters were tuned using a randomized grid search approach to minimize prediction of cross-validation error. The hyperparameter grid is shown in Table S2. The statistical metrics of the test set prediction are shown and compared with the previously reported PLS-R results in Table 4. The PLS-R model from the previously work utilized 10 principal components and the application of Savitzky–Golay filter.^14^

**Table 4:** Comparison of IRCB-XGB and SVG-PLS-R

iterative regression of corrective baselines (IRCB) and extreme gradient boosting (XGB)					
test set metrics	kyanite	wollastonite	olivine	silica	zircon
coefficient of determination (*R*^2^)	0.901	0.849	0.614	0.855	0.916
mean absolute error (g kg-solvent^–1^)	6.03	9.2	5.8	17.66	2.12
root-mean-squared error (g kg-solvent^–1^)	7.69	11.53	7.7	21.59	3.12
mean percent error	17.9	29.6	39.3	21.7	15.8

partial least-squares regression (PLS-R)					
test set metrics	kyanite	wollastonite	olivine	silica	zircon
coefficient of determination (*R*^2^)	0.932	0.912	0.527	0.885	0.837
mean absolute error (g kg-solvent^–1^)	4.57	5.66	5.2	13.53	2.52
root-mean-squared error (g kg-solvent^–1^)	6.04	7.88	6.95	17.29	3.68
mean percent error	16.5	16.7	39.4	18.2	21.4

As shown in Table 4, IRCB-XGB was generally comparable with PLS-R for the reported statistical metrics. The five IRCB-XGB models were compared with the previously reported PLS-R models using an elliptical joint confidence region (EJCR) test,^45,46^ and the results are shown in Figure S4. The IRCB-XGB model outperformed the PLS-R model for zircon and underperformed the model for wollastonite. The statistics and EJCR test for kyanite, olivine, and silica indicate slightly better performance for the PLS-R model. Overall, the statistical results for the case study 3 model indicate that using IRCB-XGB is effective for the development of regression models from complex data sets. It is plausible that the combination of IRCB and nonlinear machine learning models may require more samples for a robust calibration as compared to PLS-R. This is evidenced by the clear outperformance of ICRB-RF in case study 2 (Table S3) with several hundred calibration spectra but slightly worse overall performance in case study 3, with only around 60 training spectra for each k-fold. Alternatively, the complexity of the data or systematic error (Figure S4) may impact the comparative performance.

### IRCB Compared to Other Preprocessing Methods

The concept of the linear corrective baseline was reported in our previous work for the purpose of finding an optimal regression from two overlapping peak to develop a Raman spectroscopy model.^8^ The primary limitation of the previous approach was that only a small portion of the spectra and the single best baseline were used for a linear regression model. The previous approach did not facilitate its application to complex systems, where numerous baselines coupled with machine learning models are required to make an effective prediction on the test set. For most data sets, it is difficult to identify the region that contains the best baseline, and a single baseline is insufficient to develop a robust prediction.

Furthermore, the IRCB can be seen as an effective preprocessing tool for enhancing the RF and XGB models. IRCB was compared with other preprocessing approaches including no preprocessing (none), Savitzsky-Golay (SVG), multiplicative scatter correction (MSC), and second derivative filtering for RF (case study 2) and XGB models (case study 3). For comparison, all models were run with the default hyperparameters as shown in Table S2. The IRCB-RF and IRCB-XGB models outperformed all of the RF and XGB models that were developed with other preprocessing methods. A statistical comparison between the IRCB–machine learning models and the machine learning models with other preprocessing methods is shown in the Supporting Information Tables S5 and S6 for case study 2 and case study 3, respectively. For many of the case study 3 models, XGB showed low predictive power without the prior application of IRCB (Table S6). For example, comparing IRCB-XGB with the next best XGB modeling result, it is shown that the test set *R*^2^ was improved from 0.389 (SVG) to 0.909 (IRCB) for zircon and 0.232 (none) to 0.838 (IRCB) for silica (Table S6). For case study 2 (Table S5), the largest improvement was for the carbon model, where the test set *R*^2^ was increased from 0.639 (RF with no processing) to 0.880 (IRCB-RF).

Overall, the results of the three case studies indicate that the IRCB is a highly automatable and effective model in producing linear predictors of **Y**. However, certain challenges persist in the end-to-end automation of this model development process. Although IRCB may be utilized to automatically select the spectral regions with the highest importance for **Y**, the developer is still required to select the machine learning model (ELR, RF, XGB), determine the threshold percentage of **X**_**transform**_ to be included in **X_f_**_**eatures**_, and in some cases, manually tune the machine learning hyperparameters to avoid overfitting.

## Conclusions

We have introduced a new framework for the development of spectroscopic models that can, in some instances, outperform the existing methodologies. Generally, the matrix transformation employed within IRCB is both an effective preprocessing strategy for machine learning and a highly versatile model for generating linear features from continuous data. IRCB as a preprocessing treatment can significantly improve the application of nonlinear machine learning models RF and XGB. The efficacy of using the resulting areas from thousands of corrective baselines to improve the regression prediction with several different machine learning models further indicates the broad utility of IRCB. By applying IRCB, the optimal baseline regions can be directly mapped and identified even by a nonexpert or in instances when the physical structure of the target is unknown. For simple systems, the IRCB can capture clear molecular selectivity based on classical spectroscopic interpretation. However, the spectral regions selected by IRCB are frequently nonintuitive and difficult to manually identify for complex mixtures. The selection of certain baseline regions may provide insights into spectral interpretability that were previously difficult to identify.

The development of a feature linearization and extraction technique that does not rely upon user experience represents a key milestone toward the automation of chemometric regression analysis. The removal of variable preprocessing requirements can significantly lower the barrier of entry to model development. The proposed model may help to democratize accessibility to the development process, as facilitated by a more structured and scientific approach. The ongoing efforts in place to modify IRCB for application to classification problems are primarily focused on new functions for selecting the most relevant features from **X**_**transform**_. Furthermore, it is important to investigate strategies that optimize the threshold percentage of features that are included in the regression model, can determine which machine learning predictive model is most appropriate, and can automatically tune the machine learning hyperparameters to avoid overfitting.

## Data Availability

Data analysis was performed in Python (v 3.9). The Python modules we developed to run these case studies, a complete list of package dependencies, and the case study raw data (.xlsx format) are available from https://github.com/mglacier/IRCB. FTIR spectra were collected in ICIR (v 7.1, Mettler Toledo).

## Supporting Information Available

Key python functions; HPLC method information (Figure S1 and Table S1); case study 2 feature inclusion (Figure S2); case study 3 regression plots (Figure S3); EJCR test (Figure S4); hyperparameter information (Tables S2 and S4); case study 2 benchmark (Table S3); and preprocessing comparison (Tables S5 and S6) (PDF)
Every baseline in the **X_features_** for each analyte for the three case studies (XLSX)
