# Supplementary material for: Iterative Regression of Corrective Baselines (IRCB): A New Model for Quantitative Spectroscopy
Source: J Chem Inf Model. 2024 Jun 19;64(13):5006–15. doi: 10.1021/acs.jcim.4c00359 (PMC11234360; doi:10.1021/acs.jcim.4c00359)
Supplement: Supplementary file 1 — ci4c00359_si_001.pdf [file ci4c00359_si_001.pdf]

Supporting Information for "Iterative Regression of Corrective Baselines (IRCB): A New Model for Quantitative Spectroscopy"

Matthew Glace[1], Roudabeh S. Moazeni-Pourasil[2], Daniel W. Cook[2], Thomas D. Roper[1]*

1. Department of Chemical and Life Sciences Engineering, Virginia Commonwealth University, Richmond, VA 23284

2. Medicines for All Institute, Virginia Commonwealth University, Richmond, VA 23284

Abbreviations

2-IP: 2-isopropyl phenol
CV- Cross Validation
ELR: Ensemble Linear Regression
FDA: Food and Drug Administration
FTIR: Fourier Transform Infrared
HDIPBA: 4-hydroxy-3,5-diisopropylbenzoic acid
HPLC: High Performance Liquid Chromatography
ICH: International Counsel for Harmonization
IR: Infrared
IRCB: Iterative Regression of Corrective Baselines
LOOCV: Leave One Out Cross Validation
MSC: Multiplicative Scatter Correction
NIR: Near Infrared
PAT: Process Analytical Technologies
PLS-R: Partial Least Squares Regression
RF: Random Forest
SVG: Savitzky–Golay
XGB: Extreme Gradient Boosting

Python Functions for Key Operations (Ref. Figure 1)

Key Operation 1: $\mathbf{X}_{\mathbf{Transform}}$ Generation

Input Variables:

'data': $\mathbf{X}_{\mathbf{Calibration}}$ with wavenumber label as the first column (optionally, generic **X** for combined training/testing set, also with wavenumber label as the first column)
'max_range': the maximum permissible length of a baseline
- typically set > pixel count to generate all possible combinations
- lowering decreases computational time but generally decreases solution quality

Output Variables:

'cfm': Transposed $\mathbf{X}_{\mathbf{Transform}}$
'cfm_label: the start and stop baseline locations for each $\mathbf{X}_{\mathbf{Transform}}$ entry (zero-indexed

```
import numpy as np

# Main Function for generating X_Transform Matrix
def feature_matrix(data, max_range):

    # Generate all combinations of start and stop indices for baseline correction
    pixel_count = data.shape[0]
    ll_start_arr = np.arange(0, pixel_count-2)
    combinations = [(A, B) for A in ll_start_arr for B in range(A+2,pixel_count)]
    valid_combinations = []
    for i, (start, stop) in enumerate(combinations):
        if stop - start <= max_range:
            valid_combinations.append(i)

    # Segments data for each valid combination of start and stop indices
    cfm = np.zeros((len(data[0,:])-1, len(valid_combinations)))
    for i, j in enumerate(valid_combinations[:]):
        start, stop = combinations[j]
        data_slice = data[start:stop+1, 1:]
        cfm[:, i] = compute_area(data_slice)

    # Store start and stop indices, and range for each combination (for test set index)
    fm_label = np.zeros((2, len(valid_combinations)))
    fm_label[0] = [combinations[i][0] for i in valid_combinations]
    fm_label[1] = [combinations[i][1] for i in valid_combinations]

    return cfm, fm_label


# Performs Baseline Correction, Subtraction and Area Summation
def compute_area(data_slice):

    num_points = len(data_slice)
    interpolation_factor = np.linspace(0, 1, num_points)
    baseline = data_slice[0] + (data_slice[-1] - data_slice[0]) * interpolation_factor[:, np.newaxis]
    corrected_data = data_slice - baseline

    return np.sum(corrected_data, axis=0)
```

Key Operation 2: Ranking $X_{Transform}$ Baselines

Input Variables:

‘cal_fm: training portion of cfm (after row-wise test/train cfm split if using generic **X** from operation 1, otherwise = cfm)

‘y_cal: $\mathbf{Y}_{\mathbf{Calibration}}$ (corresponding to cal_fm)

‘criteria’: statistical metric to assigned to baseline

‘n_jobs’: number of computational cores to send jobs to (defaults to all)

Output Variables:

‘fit’: a single row array that contains a statistical metric (1-R^2) for each baseline (column-wise correspondence to cal_fm)

```
import numpy as np
from joblib import Parallel, delayed
from sklearn.linear_model import LinearRegression
from sklearn.metrics import r2_score

#Must be imported to thread jobs effectively
def regression_fit(cal_fm, y_cal, criteria = 'R2', n_jobs = -1):

    fit = np.zeros((1, len(cal_fm[1, :])))
    y_cal = y_cal.reshape(-1, 1)

    if criteria == 'R2':

        def calculate_fit(i):
            x = cal_fm[:, i].reshape(-1,1)
            model = LinearRegression().fit(x, y_cal)
            model_pred = model.predict(x)
            r2 = r2_score(y_cal, model_pred)
            return 1 - r2

    iterations = len(cal_fm[0, :])
    fit[0, :] = Parallel(n_jobs=n_jobs)(delayed(calculate_fit)(i) for i in range(iterations))

    return fit
```

fit is used to sort cal_fm/cfm, cfm_label before applying cutoff threshold to make $\mathbf{X}_{\mathbf{Features}}$

<u>High Performanace Liquid Chromatography</u>

- Isocratic, 60/40 Acetonitrile/0.1% H_3PO_4 in 18Ω H_2O
- 8 min run time, 2 min post time
- 2 mL/min
- 1 µL injection volume
- Eclipse XBD-C18 (4.6 x 250 mm; 5 µm), 30°C

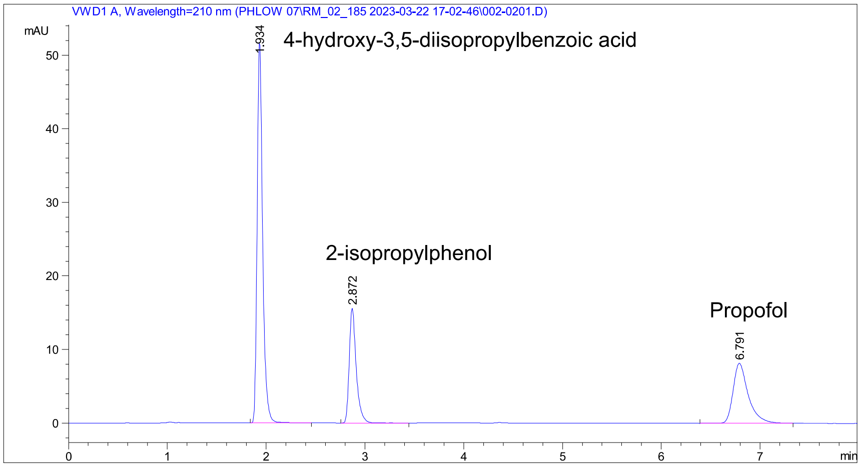

Figure S1: Sample HPLC Chromatograph

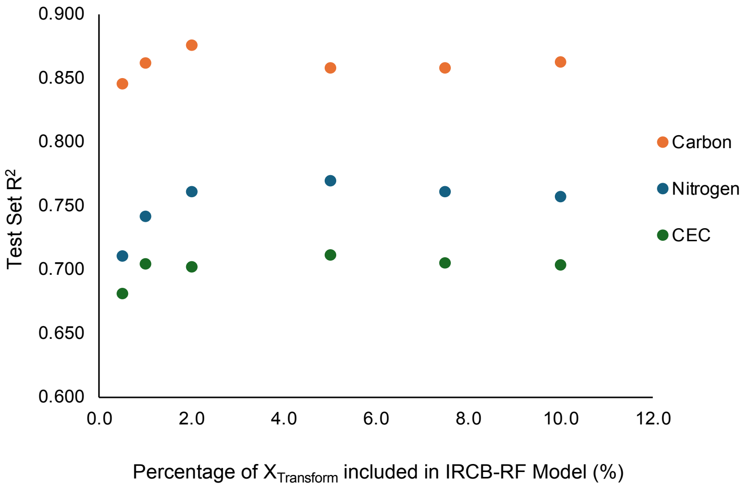

Figure S2: Case Study 2: Percentage of $X_{Transform}$ included in the IRCB-RF model vs. Test Set R^2. Observed deviations (±0.015 R^2) in the Test R^2 values from those presented in Table 3 can be attributed to variations in hardware architecture and the inherent non-determinism associated with parallel computing processes. Default hyperparameters utilized as shown in Table S2.

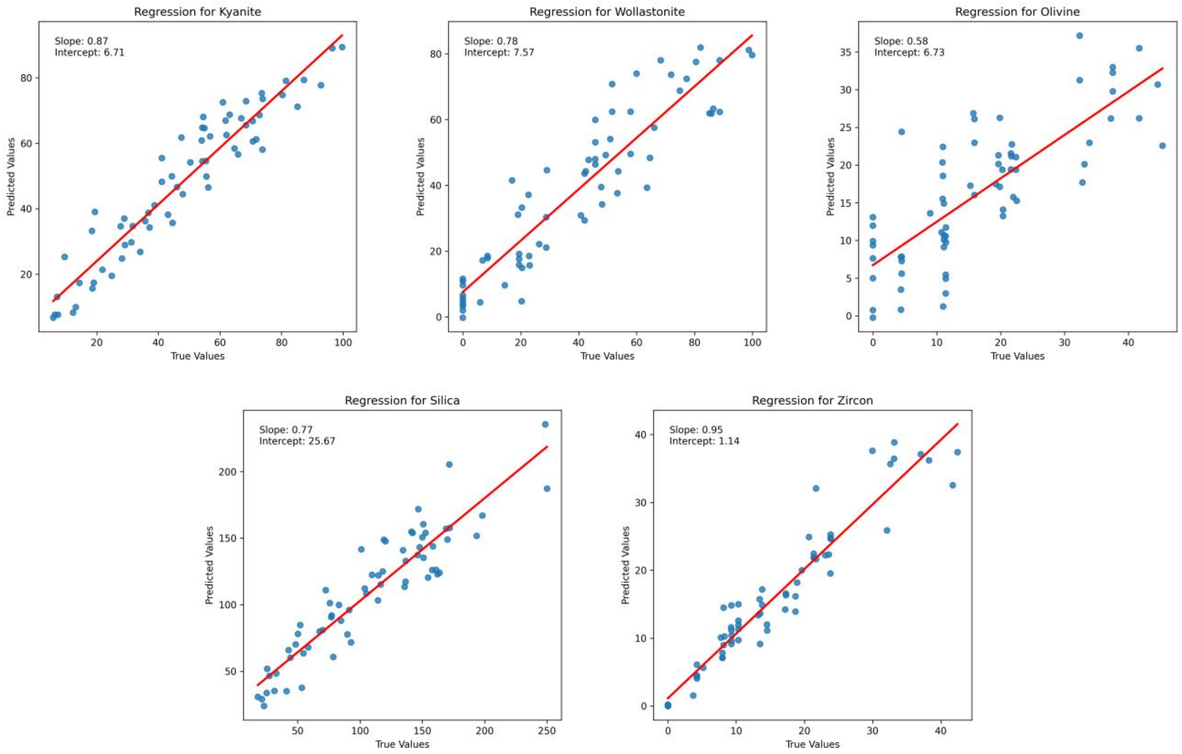

Figure S3: Regression Plot of Case Study 3

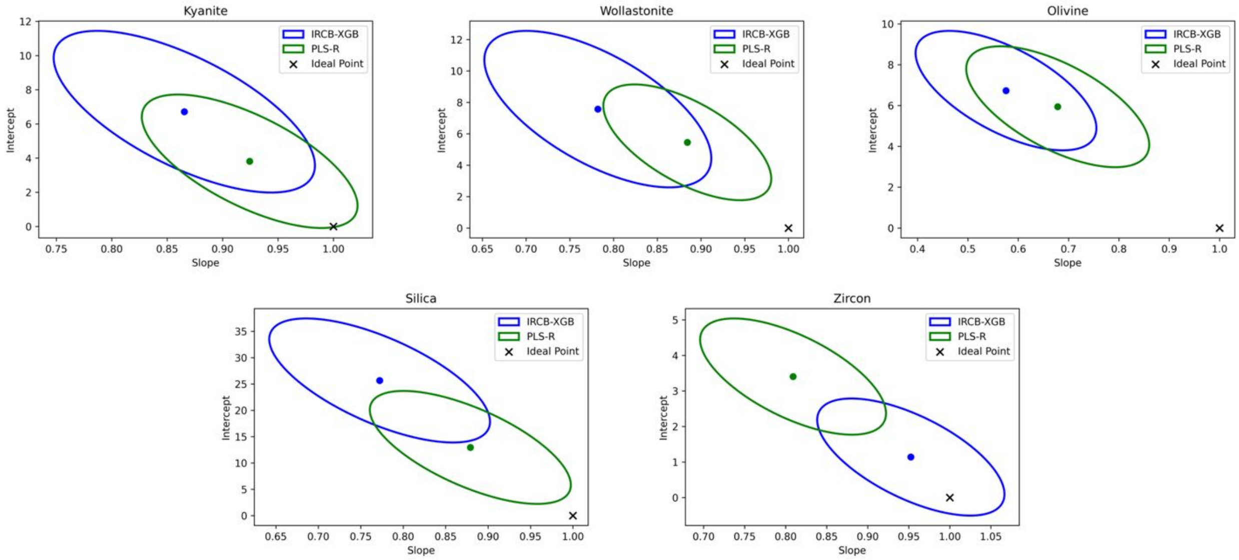

Figure S4: Elliptical Joint Confidence Region (EJCR) Test for Case Study 3

Table S1: HPLC Measurements of Case Study 1

Calibration Sample	Calibration (mg/mL)			Test Sample	Test (mg/mL)		
	Propofol	HDIPBA	2-IP		Propofol	HDIPBA	2-IP
C-1	43.2	5.2	36.8	T-1	30.0	40.0	21.9
C-2	15.6	10.1	26.2	T-2	14.9	35.6	5.7
C-3	31.6	28.7	33.1	T-3	16.2	12.1	11.7
C-4	21.5	39.4	21.5	T-4	6.5	29.4	18.9
C-5	27.6	24.8	41.9	T-5	35.9	8.9	26.6
C-6	37.5	19.6	15.9	T-6	31.5	26.3	17.5
C-7	5.3	14.2	10.9	T-7	20.1	11.8	37.2
C-8	11.1	34.0	5.6	T-8	11.0	15.1	40.8
C-9	10.5	12.6	33.7	T-9	39.9	11.1	31.6
C-10	39.4	16.1	10.1	T-10	31.5	25.6	20.1
C-11	7.7	22.8	26.5	-	-	-	-
C-12	10.9	30.1	12.0	-	-	-	-
C-13	16.8	8.2	26.4	-	-	-	-
C-14	35.7	40.4	26.2	-	-	-	-

Hyperparameters

Table S2: Default and Grid Hyperparameters for Random Forest and XGBoost

Hyperparameters	Random Forest (RF)	XGBoost (XGB)
Default	default_params_rf = {	default_params_xgb = {
	'n_estimators': 100,	'n_estimators': 100,
	'max_depth': 15,	'max_depth': 3
	'min_samples_split': 3	}
	'min_samples_leaf': 1	
	}	
Grid	param_grid = {	param_grid = {
	'n_estimators': [25, 50, 100],	'n_estimators': randint(5, 200),
	'max_depth': [5, 10],	'learning_rate': [0.5, 0.4, 0.3, 0.2, 0.1, 0.01],
	'min_samples_split': [6, 8],	'max_depth': randint(1, 8),
	'min_samples_leaf': [4, 6],	'min_child_weight': [1, 5, 10],
	'max_features': [0.25, 'sqrt'],	'colsample_bytree': [0.6, 0.7, 0.8, 0.9, 1.0],
	'bootstrap': [True],	'gamma': [0, 0.1, 0.2, 0.3, 0.4],
	}	'reg_alpha': [0, 0.01, 0.1, 1, 10],
		'reg_lambda': [0, 0.01, 0.1, 1, 10],
		'scale_pos_weight': [1, 5, 10],
		'max_delta_step': randint(1, 10),
		'colsample_bylevel': [0.6, 0.7, 0.8, 0.9, 1.0],
		'colsample_bynode': [0.6, 0.7, 0.8, 0.9, 1.0],
		'max_leaves': randint(1, 50),
		}

Table S3: Case Study 2 Comparison to Pierna et. al (2006) reported results. IRCB with 2% of $X_{Transform}$ Retained and Default Random Forest Hyperparameters (Table S2).

Methods		Participant 1<br>Norm + Local	Participant 2<br>Backpropagation NN	Participant 3<br>Bsplines + RBFN	Author 1<br>PLS	Author 2<br>Local	Author 3<br>LS-SVM	Glace<br>IRCB + RF
RMSEP	1-Nt	0.6537	0.7737	0.9655	0.8404	0.5853	0.5575	0.657
	2-CISCO	0.5577 (1 NaN)	0.7735	1.4846	1.1476	0.4332	0.559	0.527
	3-CEC	3.5823 (2 NaN)	4.6235	5.3609	4.6051	3.3328	3.4373	3.739
R^2	1-Nt	0.7673	0.63	0.438	0.5632	0.7979	0.8172	0.766
	2-CISCO	0.8702	0.77	0.5338	0.496	0.9216	0.8908	0.88
	3-CEC	0.7148	0.51	0.374	0.5087	0.7508	0.7294	0.715
Mean R^2		0.7841	0.6367	0.4486	0.5226	0.8234	0.8125	0.787

Table S4: Case Study 2 Results for Conservative Random Forest Hyperparameter Grid Search. RF Hyperparameters were selected by 5-Fold cross-validation and exhaustive grid search of parameters shown in Table S2.

Analyte	Units	Test Set		Calibration Set		RF Hyperparameters
		RMSE	R^2	RMSE	R^2	
Nitrogen	g kg-solvent^{-1}	0.688	0.734	0.315	0.929	'bootstrap': True,<br>'max_depth': 5,<br>'max_features': 0.25,<br>'min_samples_leaf': 4,<br>'min_samples_split': 6,<br>'n_estimators': 100
Carbon	Percent (%) in dry soil	0.640	0.823	0.621	0.888	'bootstrap': True,<br>'max_depth': 5,<br>'max_features': 'sqrt',<br>'min_samples_leaf': 6,<br>'min_samples_split': 6,<br>'n_estimators': 50
CEC	meq 100g^{-1}	3.615	0.746	2.154	0.892	'bootstrap': True,<br>'max_depth': 10,<br>'max_features': 0.25,<br>'min_samples_leaf': 4,<br>'min_samples_split': 6,<br>'n_estimators': 100

Table S5: A Comparison of Preprocessing Methods for Case Study 2 Random Forest Model. All models were run with the default hyperparameters as shown in Table S2. SVG: Savitsky-Golay filter with window length = 5, order = 2. MSC: multiplicative scatter correction. IRCB: Iterative regression of corrective baseline with 2% of features retained.

Analyte	Test Set Metric	Preprocessing Method				
		None	SVG	2nd Derivative	MSC	IRCB
Nitrogen	RMSE (mg ml^{-1})	0.896	0.976	1.183	1.016	0.657
	R^2	0.507	0.416	0.135	0.421	0.766
Carbon	RMSE (mg ml^{-1})	0.942	1.169	1.201	1.179	0.527
	R^2	0.639	0.434	0.377	0.542	0.880
CEC	RMSE (mg ml^{-1})	4.947	4.085	6.458	7.280	3.739
	R^2	0.421	0.605	0.050	0.143	0.715

Table S6: A Comparison of Preprocessing Methods for Case Study 3 XGBoost Model. All models were run with the default hyperparameters as shown in Table S2. SVG: Savitsky-Golay filter with window length = 5, order = 2. MSC: multiplicative scatter correction. IRCB: Iterative regression of corrective baseline with 2% of features retained.

Analyte	Test Set Metric	Preprocessing Method				
		None	SVG	2nd Derivative	MSC	IRCB
Kyanite	RMSE (g kg $solvent^{-1}$)	19.543	19.183	25.598	19.543	8.572
	R^2	0.409	0.433	0.040	0.409	0.877
Wollastonite	RMSE (g kg $solvent^{-1}$)	19.052	19.075	20.008	19.052	13.463
	R^2	0.572	0.571	0.541	0.572	0.787
Olivine	RMSE (g kg $solvent^{-1}$)	9.233	10.212	12.835	9.233	8.183
	R^2	0.457	0.361	0.022	0.457	0.560
Silica	RMSE (g kg $solvent^{-1}$)	51.657	52.984	60.716	51.657	22.563
	R^2	0.232	0.203	0.006	0.232	0.838
Zircon	RMSE (g kg $solvent^{-1}$)	8.817	8.382	9.371	8.817	3.239
	R^2	0.330	0.389	0.232	0.330	0.909
